# Region-specific vulnerability in neurodegeneration: lessons from normal ageing

**DOI:** 10.1016/j.arr.2021.101311

**Published:** 2021-05

**Authors:** Virenkumar A. Pandya, Rickie Patani

**Affiliations:** a Department of Neuromuscular Diseases, University College London Queen Square Institute of Neurology, Queen Square, London, WC1N 3BG, UK; b The Francis Crick Institute, 1 Midland Road, London, NW1 1AT, UK

**Keywords:** Ageing, Alzheimer’s disease, Parkinson’s disease, Amyotrophic lateral sclerosis, Region-specific, Selective vulnerability

## Abstract

A number of age-associated neurodegenerative diseases, including Alzheimer’s disease (AD), Parkinson’s disease (PD) and amyotrophic lateral sclerosis (ALS), possess a shared characteristic of region-specific neurodegeneration. However, the mechanisms which determine why particular regions within the nervous system are selectively vulnerable to neurodegeneration, whilst others remain relatively unaffected throughout disease progression, remain elusive. Here, we review how regional susceptibility to the ubiquitous physiological phenomenon of normal ageing might underlie the vulnerability of these same regions to neurodegeneration, highlighting three regions archetypally associated with AD, PD and ALS (the hippocampus, substantia nigra pars compacta and ventral spinal cord, respectively), as especially prone to age-related alterations. Placing particular emphasis on these three regions, we comprehensively explore differential regional susceptibility to nervous system tissue, cellular and molecular level ageing to provide an integrated perspective on why age-related neurodegenerative diseases exhibit region-selective vulnerability. Combining these principles with increasingly recognised differences between chronological and biological ageing (termed differential or ‘delta’ ageing) might ultimately guide therapeutic approaches for these devastating neurodegenerative diseases, for which a paucity of disease modifying and/or life promoting treatments currently exist.

## Highlights

• Why neurodegenerative disease pathology is regionally restricted remains elusive.
• Regions selectively prone to neurodegeneration are also vulnerable to normal ageing.
• Nervous system tissue, cellular and molecular ageing may determine regional vulnerability.
• Differential ageing can conceptually extend from an individual to subcellular scale.
• An understanding of region-specific vulnerability might guide therapeutic advances.

## 1 Introduction

An inevitable consequence of an ageing and growing society is an upsurge in age-associated neurodegenerative disease prevalence. Within this category are Alzheimer’s disease (AD), Parkinson’s disease (PD) and amyotrophic lateral sclerosis (ALS), all of which ultimately debilitate patients with progressive symptoms, significantly impacting their functional capacity and quality of life. The wider impact of these devastating diseases on patients, families, carers and society is immeasurable.

AD, the most prevalent neurodegenerative disease, is characterised by impaired episodic memory, cognitive decline and behavioural changes, severely impacting performance in activities of daily living and causing widespread loss of independence beyond this. The average life expectancy from presentation is 8.5 years. The disease is underpinned by the deposition and/or spread of two key pathological substrates, namely extracellular amyloid plaques, primarily composed of aberrantly folded amyloid beta with 42 amino acids, and intracellular neurofibrillary tangles (NFTs), containing hyperphosphorylated tau protein (reviewed in Lane et al., 2018).

PD, the second most prevalent neurodegenerative disease, progressively causes motor (bradykinesia, rigidity, resting tremor) and heterogeneous nonmotor (loss of olfaction, constipation, disturbed sleep, depression) symptoms, thereby substantially impacting patient quality of life. Life expectancy varies significantly, with one meta-analysis suggesting 6.9–14.3 years from diagnosis to death, and another study showing some patients to survive for more than 20 years . Neuronal Lewy bodies, principally consisting of alpha synuclein protein aggregates, form PD’s primary pathological hallmark (reviewed in Armstrong and Okun, 2020).

Although much rarer than AD and PD, ALS is a rapidly progressive and universally fatal neurodegenerative disease, characterised clinically by the presence of both upper and lower motor neuron (MN) features (including but not limited to muscle weakness, wasting, fasciculations and hyper-reflexia). Patients relentlessly lose the ability to eat, speak, locomote and ultimately breathe, giving ALS a prognosis of 2–5 years from diagnosis to mortality, worse than AD and PD. The majority of patients possess neuronal inclusions positive for the RNA binding protein TDP-43 (reviewed in Van Damme et al., 2017).

**Fig. 1:**
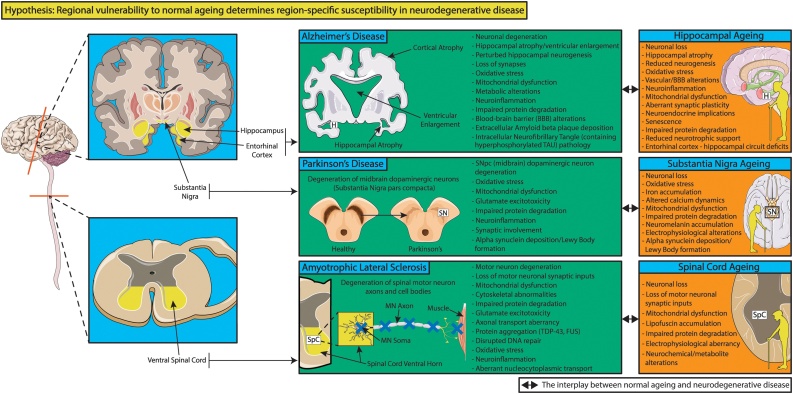
Age-related neurodegenerative diseases display region-specific pathological patterns. Particular regions of the nervous system are selectively susceptible to degenerative decline in Alzheimer’s disease (AD), Parkinson’s disease (PD) and amyotrophic lateral sclerosis (ALS), however the mechanisms establishing the regionally restricted nature of age-related neurodegenerative diseases remain undefined. Indeed, regional pathological involvement of the hippocampus, substantia nigra pars compacta and ventral spinal cord determines major clinical and personal manifestations of AD, PD and ALS respectively, with important consequences for quality of life, healthspan and/or lifespan. There is an interplay between normal regional ageing and neurodegenerative disease pathology. Whether certain regions, such as the hippocampus, substantia nigra pars compacta and ventral spinal cord are more susceptible to age-associated alterations than other nervous system regions, which in turn renders them prone to disease-specific pathology, is reviewed in the main text. H = hippocampus; SN(pc) = substantia nigra (pars compacta); SpC = spinal cord. MN = motor neuron. Templates used/adapted to create this figure are freely available from Servier Medical Art (https://smart.servier.com/). (References used to create this figure: Bettio et al., 2017; Branch et al., 2014; Cenini and Voos, 2019; De Strooper and Karran, 2016; Hindle, 2010; Keller et al., 2000; Mu and Gage, 2011; Pandya and Patani, 2019; Penney et al., 2020; Piekarz et al., 2020; Reagh et al., 2018; Reeve et al., 2014; Trist et al., 2019; Van Damme et al., 2017).

Despite clinical and pathological differences, AD, PD and ALS share the attribute of stereotyped pathology in specific brain regions whilst leaving others relatively unaffected (Braak et al., 2006, 2003; Brettschneider et al., 2013). Indeed, NFTs are detected early in the entorhinal cortex and hippocampus during AD pathogenesis (Braak et al., 2006; reviewed in Lane et al., 2018). Lewy pathology is earliest detected in the medulla oblongata and olfactory bulb, in line with some of PD’s pre-motor symptoms, however subsequent pathological involvement of the midbrain, notably the substantia nigra pars compacta (SNpc) is associated with classical parkinsonian motor symptoms (reviewed in Armstrong and Okun, 2020; Braak et al., 2003). Similarly, early phosphorylated TDP-43 pathology is noted in the ventral spinal cord, lower brainstem and frontal cortex in ALS (reviewed in Braak et al., 2013; Brettschneider et al., 2013). Why the hippocampus, SNpc and ventral spinal cord are particularly prone to AD, PD and ALS respectively, whilst other regions are relatively spared, remains elusive (Fig. 1).

The universal physiological phenomenon of normal ageing is the largest risk factor for AD, PD and ALS. Indeed, ageing imposes an array of molecular, cellular, structural and functional modifications across the nervous system, including in the aforementioned regions that are most susceptible to neurodegeneration (Fig. 1). Given the interplay between mechanisms of normal ageing and AD (reviewed in Xia et al., 2018), PD (reviews: Collier et al., 2017; Hindle, 2010) and ALS (reviewed in Pandya and Patani, 2019), it is possible that disease-prone regions are also uniquely susceptible to normal ageing, which forms the subject matter of this review.

Specifically, we review whether the hippocampus, SNpc and ventral spinal cord are nervous system regions particularly vulnerable to age-related alterations which in turn might contribute to their susceptibility to AD, PD and ALS respectively. A comprehensive understanding of the mechanisms behind regional susceptibility/resistance to neurodegenerative pathology might ultimately guide development of targeted disease modifying therapies for patients, which are currently scarce and desperately required.

## 2 Differential susceptibility to ageing: individuals to organs

The notion of differential susceptibility to ageing has been demonstrated at an organismal level, with some individuals biologically ageing better and others worse than expected for their true chronological age (reviewed in Khan et al., 2017; Rhinn and Abeliovich, 2017). Indeed, for a given chronological age, an individual might be characterised as an ‘accelerated ager’ (biological > chronological age), ‘normal ager’ (biological = chronological age) or ‘super ager’ (chronological > biological age) (reviewed in Khan et al., 2017).

Differential/delta ageing quantifies the difference between an individual’s apparent biological age derived from a given age-associated phenotypic trait and their true chronological age (Rhinn and Abeliovich, 2017). TMEM106B and Progranulin were identified as genetic determinants of delta ageing in the frontal cortices of older individuals, contributing to their heterogeneous rates of cortical ageing. It seems therefore that certain individuals might be more susceptible to the effects of ageing than their counterparts. Moreover, it is possible that the concept of differential ageing is maintained at the organ, cellular and even intracellular level.

The trajectories of age-associated functional decline vary significantly between different organ systems, indicating heterogeneous organ sensitivity to ageing (reviewed in Khan et al., 2017). The cardiovascular and neurological systems for example are relatively more affected by biological ageing than the gastrointestinal system, which only undergoes modest functional decline over time. Hence, both individuals and their organs are differentially prone to age-related decline.

Recently, individuals have been suggested to possess distinct ageing rates and patterns, termed ageotypes, where certain biological pathways are enriched and consequently stronger associated with their ageing than others (Ahadi et al., 2020). These identified pathways were further combined into 4 major age-associated pathways (kidney, liver, immune and metabolic dysregulation), and their relative ageing contribution compared. For example, one individual showed relatively robust age-association in the kidney dysfunction pathway, but only minor alterations in the remaining 3 pathways; other individuals aged in all 4 pathways. With a number of genetic, environmental and lifestyle influences potentially contributing to an individual’s ageotype, it seems that the effects of normal ageing on an individual/pathway level might be rather personalised.

**Fig. 2:**
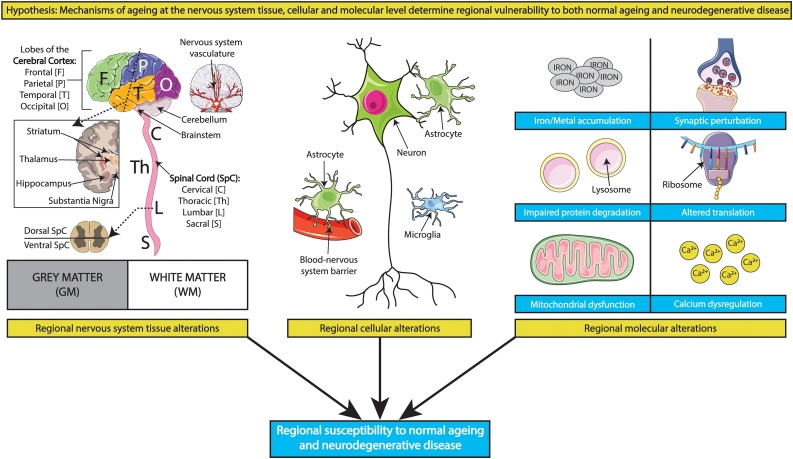
An integrated multi-scale perspective of regional ageing and disease susceptibility. A variety of age-associated mechanisms at the nervous system tissue, cellular and molecular levels might determine regional vulnerability to ageing, and in turn partially explain why age-related neurodegenerative disease pathology affects certain regions whilst leaving others relatively unaffected. Some contributory mechanisms at these levels are depicted above but reviewed comprehensively in the main text. Templates used/adapted to create this figure are freely available from Servier Medical Art (https://smart.servier.com/).

The principle of differential susceptibility to ageing observed in organisms and organ systems might further directly translate to distinct regions within organs. Focussing here within the brain and spinal cord, a number of potential mechanisms exist at the nervous system tissue, cellular and molecular levels which might render particular regions more vulnerable to ageing than their relatively unaffected counterparts (Fig. 2). These mechanisms, as well as their possible disease implications, are discussed in detail below.

## 3 Nervous system tissue level determinants of region-specific vulnerability

### 3.1 Grey and white matter ageing

Regions within the brain and spinal cord are heterogeneously composed of grey matter (GM; enriched in neuronal somas) and/or white matter (WM; primarily myelinated axons). Neuroimaging data have suggested that GM and WM age differently, with GM volume displaying a constant, linear decline over the adult lifespan, whereas WM volume followed a quadratic trajectory with age, increasing in early adulthood and then reducing from middle age onwards at a rate greater than GM volume (Ge et al., 2002). Indeed, taking a regional approach, an array of cortical and subcortical/deep regions showed linear negative correlation between GM volume and age. WM volume on the other hand showed linear negative correlations with age in certain regions, and nonlinear quadratic relationships with age in other regions (Giorgio et al., 2010), indicating differential ageing between GM and WM at the regional level.

The application of statistical methods to estimate the volumetric trajectories of 17 human brain regions across life revealed heterogeneous regional patterns, broadly categorised into linear reduction, steep, nonlinear decline and stability followed by decline (Fjell et al., 2013). The hippocampus, which displayed the latter age trajectory, possessed one of the steepest estimated declines between 60−90 years in this cross-sectional analysis. Longitudinal data from an independent sample aged 60−90 years revealed that the hippocampus shrunk at the fastest rate in this cohort. In line with this, the rate of age-associated GM volumetric decline was more rapid in the hippocampus than in other cortical/subcortical regions of individuals aged 30–99 years; of note, no significant age-related changes were detected in the SN (Jernigan et al., 2001). Moreover, hippocampal and entorhinal cortex atrophy rates were found to be increased with ageing in a longitudinal study of cognitively normal individuals (Du et al., 2006). Of 9 anatomical brain networks defined based on structural covariation of GM volume amongst healthy middle aged to older adults (45–85 years), 4 showed GM volume decreases with age, with the largest age-association in the network containing the thalamus, nucleus accumbens, caudate nucleus and hippocampus (Hafkemeijer et al., 2014). Together, these data seem to highlight the hippocampus as a region prone to age-related decline.

A longitudinal study with 7 years follow-up revealed differential susceptibility of WM fibre types to age-related changes (Bender et al., 2016). WM fibres can travel to the ipsilateral cortex (association fibres), contralateral cortex (commissural fibres) or brainstem/spinal cord (projection fibres) (reviewed in Schmahmann et al., 2008). Indeed, the decline in diffusion properties over time was more pronounced in association than commissural and projection WM fibres (Bender et al., 2016). The effects of ageing on association (cingulum, uncinate fasciculus, inferior longitudinal fasciculus), commissural (genu, splenium) and fornix WM fibre tracts were determined by diffusion tensor imaging (DTI)/quantitative fibre tracking in young and elderly individuals (Zahr et al., 2009). Results revealed an anterior-posterior distinction in susceptibility of these WM fibres to ageing, with the anterior fibre systems, namely the genu, uncinate fasciculus and fornix, more vulnerable to age than posterior systems (splenium, cingulum, inferior longitudinal fasciculus). Moreover, DTI metrics were correlated to working memory, motor performance and problem-solving functional tests. This revealed relations between working memory/problem solving and the genu and fornix and between motor performance and genu, splenium, fornix and uncinate fasciculus metrics. Interestingly, a number of these functionally important WM fibre tracts were those prone to age-related effects (anterior fibre systems). Hence, age-related compromise of regional WM fibre integrity might have important functional consequences (Zahr et al., 2009). Importantly, the age-prone fornix originates at the hippocampus, suggesting GM and WM vulnerability to ageing in this region. Indeed, fornix WM glial damage during ageing might in turn cause hippocampal GM damage, possibly rendering this region susceptible to AD pathology (Metzler-Baddeley et al., 2019). Notably, these age-related fornix/hippocampal alterations were associated with performance in episodic memory tasks.

The last in first out hypothesis of nervous system ageing proposes that the latest ontogenetically/phylogenetically developing brain regions are also those that are early to degenerate with age (Douaud et al., 2014). Indeed, later developing WM association fibres were more vulnerable than their commissural and projection fibre counterparts, in line with this hypothesis (Bender et al., 2016). Moreover, within the projection fibre group, the later developing anterior limb of the internal capsule was more prone to ageing than the posterior limb. Structural MRI in healthy individuals revealed a brain network comprising primarily of transmodal, limbic and paralimbic regions which develops late (last in) and shows accelerated degeneration in age (first out) compared to other GM regions (Douaud et al., 2014). Intriguingly, this network spatially overlaps GM regions which undergo accelerated atrophy in AD, and indeed associated with episodic memory, in line with the hypothesis that regional vulnerability to ageing might possibly determine regional susceptibility to AD. It has been proposed that the regions that developed recently in human evolution are those selectively vulnerable to pathology in a host of neurodegenerative diseases, including AD, PD and ALS (Eisen, 2009; Eisen et al., 2014; Ghika, 2008). Whether there is a possible mechanistic interplay between the last in first out hypothesis of ageing and neurodegenerative disease requires further investigation.

### 3.2 Nervous system vascular ageing

Blood is supplied to the brain and spinal cord via a complex network of vessels deriving from the internal carotid and vertebral arteries (reviewed in Chandra et al., 2017). Regional reductions in adjusted cerebral blood flow (CBF) were noted in a number of structures within the association/limbic cortices, whilst CBF in other regions did not correlate with age (Martin et al., 1991). Notably, the association/limbic regions selectively displaying reduced adjusted CBF with age here are functionally intertwined with cognition and memory (Martin et al., 1991), clinical measures which are also impaired in many neurodegenerative diseases. Additionally, region-selective age-related CBF reductions in healthy adults were uncoupled from regions displaying GM volume loss/cortical thinning with age; some regions with substantial cortical thinning during ageing had concurrent preserved CBF, including the hippocampus (Chen et al., 2011). Arterial elasticity, on the other hand, was revealed as a mediator of the relationship between ageing and cortical volume. Moreover, differential regional susceptibility to ageing was noted for both volume and arterial stiffness, with intraindividual analysis revealing that regions possessing low arterial elasticity match those with small cortical volumes (apparent primarily in older adults [>48 years]) (Chiarelli et al., 2017). One can speculate whether this region-specific vulnerability to vascular alterations during normal ageing, with its possible clinical implications, might play a role in region-selective neurodegenerative diseases. Indeed, regional CBF reductions have been noted in AD, PD and ALS, as well as a host of other vascular perturbations (reviewed in Sweeney et al., 2018). In cognitively normal elderly individuals (58–87 years), the presence of subcortical lacunes (small infarcts) increased the atrophy rate of the hippocampus but not the entorhinal cortex in addition to age (Du et al., 2006). This finding was suggested to correspond with the hippocampus being a region selectively vulnerable to ischaemia/hypoxia. Moreover, there was no noticeable association between hypertension and atrophy rates in either the hippocampus or entorhinal cortex. Altogether, these findings indicate that vascular aberrancy might somewhat selectively accelerate hippocampal decline with ageing, possibly contributing to making it a region prone to neurodegenerative disease.

Age-related vessel loss displayed regional heterogeneity in a study of 15 mouse brain regions, with WM (relative to GM) regions and those supplied by the anterior cerebral arteries particularly prone (Schager and Brown, 2020). The greatest vascular loss was noted in the corpus callosum, with a number of cortical regions also showing significantly reduced vessel length with ageing. The hippocampus was highlighted as a subcortical region showing significant vessel length reduction, with the SN pars reticulata showing a trend decrease; other regions such as the thalamus showed no changes. Notably, ageing also affected vessel tortuosity and diameter in a region-specific manner, but such changes were not predictive of vessel loss. Regional variations were also seen in the presence of vessel sparse zones, which were particularly frequent in the corpus callosum and SN pars reticulata. Indeed, vulnerability of regions to vessel loss in ageing might be partially predicted by their susceptibility to longterm capillary obstructions and inability to clear them. It is possible that region-specific vulnerability to age-associated vascular changes might have functional importance in cognitive decline, given vessel susceptibility in the corpus callosum, limbic cortex and hippocampus for example (Schager and Brown, 2020). Whether vascular alterations in the SNpc mimic those studied in the SN pars reticulata here requires further investigation.

In ageing rat spinal cords, the number of central arteries (derived from the ventral spinal artery) per unit length of cord decreased with age, also showing increased tortuosity (Qiu and Zhu, 2004). Capillary density, which was higher in GM (than WM) and the anterior horn (than the posterior horn), significantly decreased with ageing in GM. A number of structural changes, including loss of elasticity, were also noted in the aged ventral spinal artery. The authors suggested that higher capillary density in the anterior compared to the posterior cord might render this region more susceptible to damage/anoxia/ischaemia. Of note, the ventral/anterior spinal cord is selectively susceptible to early ALS pathology (reviewed in Braak et al., 2013; Brettschneider et al., 2013). Despite anatomical similarities between rat and human spinal cord arterial vasculature, some notable species differences exist (Qiu and Zhu, 2004), highlighting the importance of a somewhat cautious approach when extrapolating findings between species. Altogether, the regions which are shown to be susceptible to age-related vascular perturbations might also be prone to further insults and neurodegenerative disease-specific mechanisms.

Overall, a variety of age-related alterations at the nervous system tissue level, including changes to brain and spinal cord GM, WM and vasculature, show regional heterogeneity (Table 1). Functional consequences arising from such ageing mechanisms might be intertwined with those in neurodegenerative disease. Indeed, certain regions which seemed particularly vulnerable to ageing at the nervous system tissue level, were associated with cognitive decline. Moreover, the hippocampus, pathologically implicated in AD, was associated with GM, WM and vascular mechanisms of nervous system tissue level ageing. Whether the SNpc and anterior/ventral spinal cord are equally susceptible requires further study. Regional heterogeneity to ageing might therefore have important overlaps with region-specific neurodegenerative disease pathology.

**Table 1:** Summary: Nervous system tissue level determinants of region-specific vulnerability.

Archetypal neurodegenerative disease-associated region	Nervous system tissue level mechanisms of regional ageing/susceptibility to neurodegenerative disease	**Key references**
Hippocampus	One of the steepest age-related volumetric declines between 60−90 years	Fjell et al., 2013
	Relatively higher regional rate of age-related grey matter volumetric decline	Jernigan et al., 2001
	White matter fibres in the fornix are susceptible to ageing	Zahr et al., 2009
	Cortical thinning but preserved cerebral blood flow	Chen et al., 2011
	Ageing/presence of subcortical lacunes increases hippocampal atrophy rate	Du et al., 2006
	Age-related vessel length reduction in the hippocampus	Schager and Brown, 2020

Substantia Nigra	No significant age-related grey matter volumetric change	Jernigan et al., 2001
	SN pars reticulata showed an age-associated trend reduction in vessel length and particularly frequent vessel sparse zones	Schager and Brown, 2020

Spinal Cord	Number of central arteries reduced on ageing, with increased vessel tortuosity	Qiu and Zhu, 2004
	Higher capillary density in the ventral spinal cord may render this region susceptible	

## 4 Cellular determinants of region-specific vulnerability

### 4.1 Neural progenitors and neurons

There exists substantial variation in the cellular composition of different nervous system regions, including regional heterogeneity in their constituent neuronal types. The specific characteristics and differential vulnerability of different neuronal subtypes to ageing or neurodegeneration might in turn render their region of occupancy, and indeed innervation, prone to insult. Hence, the principle of selective neuronal vulnerability (reviewed in Mattson and Magnus, 2006) might in fact govern regional susceptibility to ageing and disease. Below, we focus on the unique and/or pertinent characteristics of ageing hippocampal, SNpc and ventral spinal cord neurons, which might render them more vulnerable than neurons from other nervous system regions.

Physiologically, continued adult neurogenesis is regionally restricted to the subventricular zone of the lateral ventricles and subgranular zone of the hippocampal dentate gyrus (DG) (reviewed in Ming and Song, 2011), although some noncanonical neurogenic sites have been identified in adult mammals (reviewed in Feliciano et al., 2015), as well as the possibility that NG2 cells might possess neurogenic potential, however this is not entirely resolved (reviewed in Kirdajova and Anderova, 2020). Hippocampal neurogenesis has vital functional implications, potentially partaking in both learning and memory (reviewed in Seib and Martin-Villalba, 2015). Indeed, normal ageing has been associated with a decline in hippocampal neurogenesis in both rodents and primates (Ben Abdallah et al., 2010; Leuner et al., 2007; reviewed in Seib and Martin-Villalba, 2015). Despite some interspecies similarities in adult neurogenesis (Knoth et al., 2010), some notable differences (reviewed in Bergmann et al., 2015) make it is especially important to determine the presence and nature of age-associated changes in human hippocampal neurogenesis. Indeed, the number of cells expressing Doublecortin (DCX), a marker associated with adult neurogenesis, declines in the human DG with ageing, as does DCX’s overlap with a number of other neurogenesis-associated markers (Knoth et al., 2010). DCX^+^ cells are however identified throughout life in the human DG (post-mortem tissue from individuals aged 1 day to 100 years) (Knoth et al., 2010).

In line with this, post-mortem analysis of individuals aged 79–99 years with clinical diagnoses of normal cognition, mild cognitive impairment (MCI) and AD has revealed identifiable profiles for proxy markers of hippocampal neurogenesis, suggesting the presence of neural progenitor cells, neuroblasts and immature neurons in these elderly individuals (Tobin et al., 2019). Interestingly, a positive association was noted between the number of DCX^+^PCNA^+^ neuroblasts and cognitive status, corroborated by the finding that MCI patients had fewer DCX^+^PCNA^+^ cells than cognitively normal individuals. As well as this, a further positive correlation between DCX^+^PCNA^+^ cells and the functional interaction of presynaptic SNAP receptor proteins (determined by the abundance of interactions between SNAP-25 and syntaxin measured in 6 brain regions) raises the possibility of negative cognitive implications resulting from reduced hippocampal neurogenesis in humans. Another post-mortem study identified no differences in hippocampal DCX^+^ or DCX^+^PSA-NCAM^+^ neuroblasts/immature granule neurons over a 65 year timespan (healthy individuals aged 14–79 years), suggesting stability of neurogenesis with ageing (Boldrini et al., 2018). There was however a decrease in quiescent neural progenitors in the anterior-middle DG with ageing, as well as an age-associated reduction in angiogenesis and potential decline in neuroplasticity, which might have functional consequences (Boldrini et al., 2018). A number of alterations associated with normal ageing might also impact hippocampal neurogenesis, including hormonal and vascular changes, inflammation, signalling differences and alterations to the cellular environment (reviewed in Seib and Martin-Villalba, 2015). Altogether, the above evidence suggests that the hippocampal neurogenic process and/or environment is particularly susceptible to ageing, which might also render the hippocampal region, where this characteristic is rather unique, selectively vulnerable to ageing.

Perturbed adult neurogenesis has been described in PD and AD (reviewed in Winner et al., 2011). Affected early in disease progression, neurogenesis might be vital in AD pathology with multiple AD-associated pathogenic molecules also able to modulate adult hippocampal neurogenesis (reviewed in Mu and Gage, 2011). Knocking down adult hippocampal neurogenesis in mice revealed a vital role for newborn neurons in spatial pattern separation, which is the ability to differentiate between spatially similar inputs to form distinct representations (Clelland et al., 2009). In two independent functional assays of spatial pattern separation, neurogenically perturbed mice were less able to distinguish stimuli presented in close proximity, but could decipher higher spatial separations (Clelland et al., 2009). Indeed, ageing has been associated with a reduced efficiency in spatial pattern separation in animal models and humans (reviewed in Holden and Gilbert, 2012), in line with reduced hippocampal neurogenesis. Moreover, it is possible that age-associated alterations in spatial pattern separation might also impair episodic memory, implicated in AD. With AD and MCI patients also evidenced to have disrupted spatial memory, the effects of ageing on hippocampal neurogenesis might have key implications for the susceptibility of this region to disease. Altogether therefore, its neurogenic ability might render the hippocampus prone to age-related and possibly even disease-associated disruption.

Differential susceptibility to age-related neuronal loss might also underlie regional vulnerability to ageing and, in turn, disease. Indeed, neuronal losses with ageing have been noted in hippocampal NeuN expressing neurons (Fu et al., 2015), pigmented and TH expressing SN neurons (Rudow et al., 2008) and lumbosacral spinal cord MNs (Jacob, 1998; Tomlinson and Irving, 1977), although the latter has been recently contested (Maxwell et al., 2018). Regional comparison of neuron number and density across the ageing mouse nervous system revealed differential patterns (Fu et al., 2015). More specifically, whilst the neocortex did not show any significant alterations in neuron number/density with ageing, hippocampal neurons showed a strong age-dependent numerical reduction between 7–25 months age, although neuronal density did not display age-dependence. Moreover, neurons in the spinal cord underwent an age-associated increase, whereas neuronal density decreased. The hippocampus was thereby highlighted as a region susceptible to age-related neuronal decline, whereas the spinal cord appeared most structurally dynamic, displaying significant age-dependent alterations in more parameters than any other region studied. The SN has also been highlighted as a region displaying levels of neuronal loss higher than various other regions (reviewed in Reeve et al., 2014). It is possible that regions where neuronal number and/or density are significantly altered during ageing might be selectively prone to age and disease-related damage.

Neurons that are selectively vulnerable to ageing tend to be of large size and possess lengthy axons (reviewed in Mattson and Magnus, 2006). Neurodegenerative disease-associated neurons, including lower MNs (ALS) and hippocampal neurons (AD) fit this description, with SN dopaminergic neurons (PD) also classed as long projection neurons (reviewed in Mattson and Magnus, 2006). In line with this phenomenon, an age-related numerical reduction was only noted in human cortical neurons with large or very large cell bodies, whereas no losses were identified in neurons with small or medium sized somas with ageing (Soreq et al., 2017). Hence, morphological features of neurons in the hippocampus, SNpc and ventral spinal cord might render these neurons and their regions selectively vulnerable to age-related decline and consequently prone to neurodegenerative disease, where large and long neurons are also implicated (reviewed in Fu et al., 2018). It is possible that the age-related absence of large neurons might be due to atrophy of these neurons rather than their loss per se, or secondary to the loss of markers used to visualise these large neurons with ageing (discussed in Soreq et al., 2017), highlighting the importance of selecting multiple markers which are specifically maintained with age and in spite of atrophy. Interestingly, when spinal cord MNs (Jacob, 1998) and SN neurons (Rudow et al., 2008) were lost during ageing, remaining neurons showed compensatory increases in neuronal size/area and hypertrophy. Whether this compensatory increase in size ultimately leaves remaining neurons and the regions in which they are found selectively vulnerable to the insults of ageing and disease requires further investigation.

Differential sensitivity of neurons to oxidative stress, a key age-associated phenomenon (reviewed in Wang and Michaelis, 2010), might also underlie heterogeneous regional susceptibility to ageing and disease. Indeed, hippocampal CA1 neurons are more vulnerable to oxidative stress than their adjacent CA3 neurons (reviewed in Wang and Michaelis, 2010). Moreover, CA1 neurons have highly demanding energy requirements compared to other neuronal types, which might render them selectively prone to glucose deprivation, hypoxia and other stressors (reviewed in Mattsson et al., 2016). Neuroimaging capturing the hippocampal subfields revealed that solely CA1 displayed age-related volumetric decline, whilst other subregions showed no age correlation (Mueller et al., 2007); whether neuronal or other CA1 vulnerabilities (discussed in Mueller et al., 2007) are responsible for this selective CA1 subfield susceptibility requires further clarification. Notably, these same CA1 hippocampal neurons are implicated in early AD pathology (reviewed in Mattsson et al., 2016), rendering their selective vulnerability potentially relevant to the age and disease-related decline of the hippocampal region where they reside.

Differential susceptibility to oxidative stress also exists amongst populations of brain dopaminergic neurons, with SNpc neurons proving more prone than their ventral tegmental area (VTA) counterparts (reviewed in Wang and Michaelis, 2010). In line with this, advancing age correlated with a reduction in TH expressing neurons in the rhesus monkey ventral SN, but not in the dorsal SN or VTA (Kanaan et al., 2007). Notably, the ventral SN is selectively vulnerable to degeneration in PD, in contrast to the resistant dorsal SN and VTA. Other factors rendering SNpc neurons more vulnerable than those in the VTA are that SNpc neurons display higher basal mitochondrial oxidative phosphorylation, operate closer to maximum respiratory capacity and produce more reactive oxygen species (ROS) than VTA neurons (Pacelli et al., 2015). Moreover, axonal arborisation, axonal mitochondrial density and ATP content were elevated in SNpc neurons compared to their VTA counterparts. Together, these characteristics rendered SNpc neurons more susceptible to cytotoxicity by various stressors. It was proposed that these basal traits might increase the susceptibility of SNpc neurons to cellular damage and alterations that occur during ageing as well as potentially determining their selective vulnerability in PD.

Given their large sizes and projection lengths, MNs would be expected to have high metabolic requirement and mitochondrial activity, resulting in exposure to higher oxidative stress levels than other neurons (reviewed in Shaw and Eggett, 2000). MNs may consequently be more susceptible to age-related perturbations in mitochondrial function, which might lead to an energy deficit in this cell type and possible toxic consequences, such as excitotoxicity (reviewed in Shaw and Eggett, 2000). Moreover, MNs undergo a number of age-associated alterations which might render them prone to further disease and ALS-related insult (reviewed in Pandya and Patani, 2019). Other explanations for spinal cord MN selective vulnerability can be derived from their comparison with oculomotor MNs, which are resistant to degeneration and relatively spared in ALS (reviewed in Nijssen et al., 2017). Spinal MNs have larger somas, more complex dendritic trees and innervate more endplates than oculomotor MNs. Moreover, resistant (oculomotor) and vulnerable (spinal) MN populations possess distinct gene/protein expression profiles, more specifically enriched expression of IGF-1 and 2, parvalbumin and Glur2 in oculomotor neurons and peripherin, dynein and MMP9 in spinal MNs (reviewed in Nijssen et al., 2017). Thereby, a combination of their unique cytoarchitecture and expression signature might contribute to spinal cord MN selective vulnerability to both age-associated decline and ALS. Given that the ventral horn of the spinal cord is rich in MNs compared to the dorsal spinal cord and majority of brain regions, its constituent neuronal subtypes might determine the susceptibility of the ventral spinal cord region to ageing and ALS.

Altogether therefore, neurons within the hippocampus, SNpc and ventral spinal cord possess an array of unique and/or prominent morphological, structural and functional characteristics which might leave these neuronal types selectively vulnerable to ageing (Table 2). It is possible that nervous system regions possessing neurons sensitive to age-associated stress are in turn themselves rendered prone to age-related decline and neurodegenerative disease.

**Table 2:** Summary: Cellular level determinants of region-specific vulnerability.

Archetypal neurodegenerative disease-associated region	Cellular level mechanisms of regional ageing/susceptibility to neurodegenerative disease	**Key references**
Hippocampus	Decline in hippocampal neurogenesis (rodents/primates)	Ben Abdallah et al., 2010 Leuner et al., 2007
	Fewer Doublecortin expressing cells in the human dentate gyrus	Knoth et al., 2010
	Reduction in quiescent neural progenitors and angiogenesis in dentate gyrus	Boldrini et al., 2018
	Regional loss of hippocampal NeuN expressing neurons and nonneuronal cells	Fu et al., 2015
	Large/long hippocampal neurons are selectively vulnerable to ageing	Mattson and Magnus, 2006
	Hippocampal CA1 neurons are selectively vulnerable to oxidative stress	Wang and Michaelis, 2010
	Relatively larger changes in astrocyte-specific gene expression with ageing	Soreq et al., 2017
	Region with relatively more astrocyte expression changes with ageing	Clarke et al., 2018
	Elevated astrocytic proinflammatory phenotype compared to other regions on ageing	Clarke et al., 2018
	Lower capacity of hippocampal astrocytes to respond to injury	Cragnolini et al., 2018
	Loss of unique microglial regional identity on ageing; reduced engagement with niche	Grabert et al., 2016
	Regionally susceptible to breakdown of blood-brain barrier	Montagne et al., 2015

Substantia Nigra	Loss of pigmented and tyrosine hydroxylase expressing neurons	Rudow et al., 2008
	Higher levels of neuronal loss than other regions	Reeve et al., 2014
	Long substantia nigra dopaminergic neurons are selectively vulnerable to ageing	Mattson and Magnus, 2006
	SN pars compacta neurons are selectively vulnerable to oxidative stress	Wang and Michaelis, 2010
	Relatively larger changes in astrocyte-specific gene expression with ageing	Soreq et al., 2017
	SN astrocytes were less protective than their VTA counterparts	Kostuk et al., 2019
	High abundance of microglia and regional vulnerability to neuroinflammatory insult	Kim et al., 2000

Spinal Cord	Most structurally dynamic with ageing in terms of neuronal number/density	Fu et al., 2015
	Loss of lumbosacral motor neurons	Tomlinson and Irving, 1977
	Large/long lower motor neurons are selectively vulnerable to ageing	Mattson and Magnus, 2006
	Motor neurons are exposed to higher oxidative stress and may be more susceptible to ageing	Shaw and Eggett, 2000
	Increase in nonneuronal cell abundance with ageing	Fu et al., 2015
	Spinal cord microglia more susceptible to ageing than their brain counterparts	Ritzel et al., 2015
	Age-related increase in blood-spinal cord barrier permeability, highest in the ventral horn	Piekarz et al., 2020

### 4.2 Glia: astrocytes and microglia

Given their abundance in the human brain (approximately equal numbers to neurons (Azevedo et al., 2009)), nonneuronal cells, including glia, have imperative roles in nervous system development, physiology and pathology. Astrocytes maintain and regulate synaptic function (recycle neurotransmitters, release gliotransmitters, modify synaptic plasticity), determine ion homeostasis (such as removal of extracellular K^+^), form a key constituent of blood-nervous system barriers and deliver nutrients to metabolically support neurons (reviewed in Allen and Lyons, 2018). Microglia, nervous system immune cells, surveil their environment to ensure protection from pathogens, regulate synaptic function and remove apoptotic neurons. Both glial subtypes have been found to significantly contribute to pathology in AD, PD and ALS (reviews: Hickman et al., 2018; Phatnani and Maniatis, 2015; Serio and Patani, 2018; Tyzack et al., 2016), further challenging traditional beliefs that neurodegenerative disease mechanisms are entirely neuron centric.

Intriguingly, nonneuronal cells have been shown to undergo differential age-associated alterations in their number and/or density depending on their region (Fu et al., 2015), revealing the possibility that nonneuronal cells in certain regions might be disproportionately prone to ageing and in turn age-related disease. Indeed, whilst nonneuronal cells in the cerebellum and neocortex displayed no age-related numerical or density changes, murine hippocampal nonneuronal cells displayed a significant decrease in number between 7 and 25 months age, whereas density changes did not display age-dependence. Additionally, although the abundance of spinal cord nonneuronal cells significantly increased with ageing, cell density showed relatively weak age-related decline. Nonneuronal cells in the spinal cord and hippocampal regions, archetypal foci for age-related neurodegenerative diseases (ALS and AD, respectively), seem to alter more with ageing than their counterparts from other regions, possibly indicating that glial cells in these regions are more susceptible to change in ageing.

#### 4.2.1 Astrocytes

Several transcriptomic studies have revealed astrocytic regional heterogeneity to ageing. In adult (4 month) and aged (2 year) mice, regional analysis of the astrocyte transcriptome (hypothalamus, cerebellum, visual cortex, motor cortex) revealed disparate region-dependent expression profiles, which altered with ageing, with the hypothalamus and cerebellum showing more genes with altered expression than the cortical astrocytes, which showed the fewest age-associated gene expression changes (Boisvert et al., 2018). Although expression of certain genes were consistently altered with ageing across regions, others showed more regionally restricted age-associated expression changes, revealing unique regional alterations in ageing astrocytes. Regionally heterogeneous astrocytic responses to ageing were also identified in human post-mortem tissue. More specifically, comparison of the frontal, temporal and occipital cortices, intralobular WM, cerebellum, thalamus, putamen, medulla, SN and hippocampus of 134 deceased individuals aged 16−102 years revealed regional differences in the number and direction of gene expression alterations with ageing, with most expression changes specific for one or a few regions and some alterations across multiple/all regions (Soreq et al., 2017). Substantial regional shifts in astrocyte-specific genes upon ageing were noted, with astrocyte-specific genes that initially separated brain regions distinctly in young samples, failing to do so to the same extent in the aged cohort, indicating enhanced interregional similarity of expression signatures upon ageing. Of note, the largest changes in astrocyte-specific gene expression with ageing were identified in the hippocampus and SN, possibly indicating that astrocytes within these regions were most susceptible to ageing. Future regional transcriptomic studies applying similar principles might determine whether ventral spinal cord astrocytes, not studied here, are also selectively prone to ageing. Altogether, the selective vulnerability of hippocampal and SN astrocytes to ageing might therefore underlie the differential susceptibility of these regions to age-related neurodegenerative disease (AD and PD, respectively).

In line with this, comparing astrocytic gene expression from the murine hippocampus, striatum and cortex throughout life (ranging from P7 to 2 years age), revealed that cortical astrocytes displayed the fewest age-related expression changes, whereas more age-induced alterations were noted in striatal and hippocampal astrocytes (Clarke et al., 2018). Comparing adult (10 week) and aged (2 year) astrocytes from the hippocampus, striatum and cortex revealed stark regional heterogeneity in the responses of these regions to age, with the hippocampus and striatum displaying more differentially expressed genes than the cortex, suggesting these regions are prone to age-related change. In response to insult, astrocytes become reactive, with their adopted phenotype dependent on the nature of injury. Indeed, lipopolysaccharide (LPS)-based neuroinflammation, likely through microglia, induces a proinflammatory phenotype, characterised by complement activation and antigen presentation (Zamanian et al., 2012). Reactive astrocytes, however, develop a neuroprotective phenotype in response to middle cerebral artery occlusion (ischaemia), inducing neurotrophic cytokines and thrombospondins. Indeed, upon ageing, astrocytes upregulated more proinflammatory genes than neuroprotective genes independent of region, indicating that ageing induces potentially more detrimental reactive transformation in these cells (Clarke et al., 2018). However, region-dependent alterations were noted in the extent of reactivity, with hippocampal and striatal astrocytes upregulating both a higher number of proinflammatory reactive genes, and displaying an increased fold induction of these genes, when compared to cortical astrocytes. Results were consistent with the notion that hippocampal and striatal astrocytes were more reactive than their cortical counterparts. Altogether, the enhanced potentially detrimental astrocyte phenotype induced by ageing in hippocampal and striatal astrocytes might render these regions susceptible to age-related damage and neurodegenerative disease; indeed, such deleterious astrocytes have been identified in AD, PD and ALS. Interestingly, astrocytic genes that were consistently upregulated at murine ages 4–12 months compared to 2 months included genes related to AD risk (*Apoe*) and PD pathogenesis (*Snca; Sncg*) (Pan et al., 2020).

By studying the responses of hippocampal, cortical and striatal astrocytes to *in vitro* scratch injury, regional variation in their wound healing capacity was noted (Cragnolini et al., 2018). More specifically, striatal astrocytes showed faster wound closure, higher proliferation rates and were the sole cells to respond to addition of the neurotrophic factor BDNF (when compared to hippocampal and cortical astrocytes). Their region-specific differential response to injury might have important implications for the capacity of astrocytes to react to nervous system damage and one can speculate that heterogeneous astrocytic ability to respond to age-related damage might also exist in a region-dependent manner, but this requires further experimental validation. Moreover, a relatively lower capacity for hippocampal astrocytes to respond to insult might render this region vulnerable to damage. Using three independent astrocytic markers (GFAP, glutamine synthetase and s100β), region-specific changes were noted between murine entorhinal cortex, hippocampal CA1 and hippocampal DG astrocytes in ageing (Rodriguez et al., 2014). Whilst hippocampal CA1 and DG astrocytes displayed age-associated increases in GFAP immunoreactive profiles, entorhinal cortex astrocytes showed a progressive age-related decline. DG and CA1 astrocytes showed age-related reductions in glutamine synthetase immunoreactivity, absent from entorhinal cortex astrocytes. S100β immunoreactive profiles increased in entorhinal cortex and DG but not CA1 hippocampal astrocytes. Altogether, ageing induced heterogeneous region-specific alterations in astrocytes, with differential changes in various astrocytic markers, which might underlie vulnerability of these regions to disease.

Regional differences in protective capacity were also identified between VTA and SN astrocytes, with various ratios (1:1, 2:1, 4:1) of SN astrocytes to SN/VTA neurons failing to protect the latter from MPP-induced toxicity (mimicking PD) (Kostuk et al., 2019). Adding 2:1 and 4:1 VTA astrocytes to SN/VTA neurons was however protective, revealing a differential intrinsic protective capacity of these astrocytes dependent on region. Indeed, VTA astrocyte conditioned media allowed greater protection for SN/VTA neurons versus MPP than media conditioned by cortical or SN astrocytes, suggesting VTA astrocytic protective secretions that were lower/absent from SN/cortical astrocytes. GDF15 was identified as a potential protective VTA astrocytic secreted factor. It is thereby possible that regional astrocytic differences underlie selective susceptibility of the SN to insult and damage in PD. Whether this phenomenon might also render the SN region prone to age-related damage and in turn PD, is of interest for future studies. Altogether, basal regional differences in astrocytic response to injury might determine the vulnerability of regions, such as the hippocampus and SN, to age and disease-associated damage.

#### 4.2.2 Microglia

Genes displaying persistently elevated expression in ageing microglia (mice 4–12 months of age vs. 2 month-old counterparts) included those involved in the cytokine pathway, immunoregulation and inflammation (Pan et al., 2020). Of note, limited expression overlap was detected between ageing mouse microglia and their human contemporaries. Similarly, although transcriptomic comparison of microglia isolated from human post-mortem tissue revealed substantial overlap with murine cortical microglia (with a few notable exceptions with much lower expression in mouse microglia), genes differentially expressed during ageing showed only limited overlap between the two species (Galatro et al., 2017). Altogether, these studies indicate that human and murine microglia age differently, highlighting the importance of supporting conclusions derived from animal models with orthogonal studies using human models of microglial ageing. Indeed, age-related human microglial gene expression changes across an array of post-mortem cortical and subcortical regions revealed that most genes increased expression levels across brain regions (Soreq et al., 2017), indicating that human microglia might not age differently dependent on their region, at least in transcriptomic terms.

Regional transcriptomic heterogeneity in microglia was however noted in mice aged 4 months, with striatal and cortical samples showing a closer relationship than the relatively more distinct hippocampal and cerebellar samples. Furthermore, gene/pathway analysis suggested that microglia residing in hippocampal and cerebellar regions are more immune-alert than their cortical/striatal counterparts (Grabert et al., 2016). Upon ageing, at 22 months, although the cerebellum remained distinct from other regional microglial populations, the hippocampal samples converged with cortical and striatal microglia, indicating possible loss of their unique regional identity with ageing. Cerebellar microglia, however, seemed to possess higher sensitivity to alteration with ageing. Most hippocampal microglial age-associated expression changes occurred late (between 12–22 months), compared to early striatal changes and relatively consistent cerebellar/cortical changes. Regional heterogeneity in response to ageing was thereby apparent in microglia, with important implications for cerebellar and hippocampal microglia, and pathway analysis suggesting that the latter possibly became disengaged with their niche in ageing (Grabert et al., 2016). Such an age-associated decline in hippocampal microglia immune environmental alertness might also render the hippocampus selectively prone to aberrance in human ageing and neurodegenerative disease.

Like astrocytes, microglia have also been shown to possess basal regional differences in response to injury. Indeed, LPS injection into the adult rat SN, cortex and hippocampus led to large reductions in NeuN^+^ neurons (and TH^+^ dopaminergic neurons) after 7 days in the subsequently dissected SN, whereas no difference was seen in hippocampal and cortical regions (Kim et al., 2000). Indeed, the SN contained the most microglia of the three regions. In mesencephalic neuron-glia cocultures, LPS stimulation induced high levels of TNFα in culture medium at 6 h post treatment, a factor predominantly released by microglia. Comparatively, low levels of TNFα were detected in LPS-treated cortical/hippocampal neuron-glia cocultures. A higher number of reactive microglia were present in LPS-treated mesencephalic cocultures. Normalising for microglial number from the three regions revealed similar levels of TNFα after LPS treatment, indicating that the increased number of activated microglia in mesencephalic cocultures might determine their increased vulnerability to LPS treatment (rather than inherent regional microglial differences). Similarly, addition of more cortical or mesencephalic microglia to cortical neuron-glia cocultures induced neuronal loss and increased TNFα after LPS stimulation (Kim et al., 2000). Hence, the heterogeneous susceptibility of regions and their neurons might be partially dependent on the number of microglia in these regions. Intriguingly, the hippocampus and SN have been highlighted as regions with particularly high numbers of microglia (in the adult mouse brain) (Lawson et al., 1990). The abovementioned regional differences in microglial response to neuroinflammatory insult might be relevant to ageing, which is itself related to inflammation (reviewed in Lopez-Otin et al., 2013).

Regionally heterogeneous microglial phenotypes were also noted in aged mice (20–21 months), with WM regions and the cerebellum displaying enhanced morphological alterations and more prominent expression increases in functional microglial markers (Hart et al., 2012). Intriguingly, more caudal regions showed greater increases in microglial marker expression, consistent with a possible rostro-caudal gradient in microglial activation. In line with this, morphological and expression changes indicating microglial activation with ageing were found at a greater extent in the murine spinal cord than the brain, despite being noted in both (Ritzel et al., 2015). Indeed, ageing spinal cord microglia displayed elevated TNFα intensities and proportions compared to ageing brain microglia. Regional differences were also noted in microglial phagocytic ability. At baseline, a number of proinflammatory cytokines were elevated in spinal cord compared to brain microglia, and the blood-spinal cord barrier showed higher permeability than its brain counterpart, findings preserved in age. Altogether, this might indicate a higher activation state of spinal cord microglia, with differences between the two blood-nervous system barrier types a potential contributory mechanism (Ritzel et al., 2015).

### 4.3 Blood-nervous system barriers

Indeed, a number of differences exist between the blood-spinal cord barrier and blood-brain barrier, with the former displaying increased permeability, reduced tight and adherence junction protein expression and decreased transporter molecules (reviewed in Bartanusz et al., 2011). Whether inherent barrier differences have the potential to render the spinal cord region and its constituent cells more vulnerable to ageing and disease requires further clarification. An age-related permeability increase in the blood-spinal cord barrier was noted in old mice (27 months), with more numerous areas of horseradish peroxidase extravasation apparent compared to young mice (2 months) (Piekarz et al., 2020). Intriguingly, these age-associated areas of extravasation were highest in the ventral horn of the spinal cord, in line with this region being specifically prone to age-related compromise in barrier function and damage resulting from exposure to toxic metabolites. Additionally, brain imaging revealed the entire hippocampus, its CA1 and DG subregions (but not CA3), to be particularly prone to early age-related blood-brain barrier breakdown in normal ageing, when compared to a number of cortical, subcortical and WM regions, with injury to pericytes a potential mechanism (Montagne et al., 2015). Indeed, with further blood-brain barrier impairment in MCI patients, it is possible that this phenomenon bears important functional consequences for cognition. Regionally age-compromised blood-nervous system barrier function may thereby underlie differential regional susceptibility to ageing and disease. With the blood-brain barrier cellular constituents (astrocytes, pericytes and endothelial cells) vulnerable to age-associated change (reviewed in Valles et al., 2019), region-dependent age-related alterations at the cellular level might determine barrier compromise and its consequences in ageing and disease. Indeed, regional blood-nervous system barrier disruption has been identified in AD, PD and ALS (Garbuzova-Davis et al., 2007; reviewed in Sweeney et al., 2018).

In summary, the hippocampus, SN and spinal cord are evidenced above as regions particularly susceptible to astrocytic, microglial and blood-nervous system barrier alterations in ageing (Table 2). It is possible that normal ageing of the nonneuronal cellular constituents of these regions determines their susceptibility to neurodegenerative disease pathology.

## 5 Molecular level determinants of region-specific vulnerability

As well as age-associated phenotypes at the individual, nervous system tissue and cellular level which might determine selective regional vulnerability, an array of intracellular alterations at the molecular level might also render specific regions vulnerable to ageing and disease. These are discussed below.

Iron possesses numerous essential responsibilities within the nervous system, partaking in oxygen transport, mitochondrial oxidative phosphorylation and in the synthesis of DNA, myelin and neurotransmitters (reviewed in Ward et al., 2014). Consequently, successful iron homeostasis is imperative. Regional iron accumulation during normal ageing has been identified in human post-mortem brains, revealing the putamen, globus pallidus and caudate nucleus as regions with the most significant correlations between iron levels and age of the 14 regions examined (Ramos et al., 2014). In line with this, a neuroimaging study comparing regional iron levels between young (21–29 years) and elderly (64–86 years) participants identified higher iron concentrations in the putamen, globus pallidus, red nucleus and SN in the older group, an observation reversed in other regions (thalamic iron levels were lower in the elderly) (Bilgic et al., 2012). Moreover, a human post-mortem study examining iron dynamics in the SN and locus coeruleus during ageing (samples aged 14–97 years) revealed linear increase in iron concentration in the ageing SN, absent from the locus coeruleus, where iron concentration was much lower and remained constant (Zecca et al., 2004). The concentration of neuromelanin, an important iron chelator, increased in both regions, with a steeper increase in the SN. SN neuromelanin possessed a higher iron content than its locus coeruleus counterpart, in line with more free iron in SN neurons. Given that excessive iron accumulation is linked to neurotoxicity and neurodegeneration via possible mechanisms of oxidative stress, mitochondrial aberrance and protein aggregation (reviewed in Ward et al., 2014), age-related regional iron accumulation in the SN might render this region prone to neurodegenerative disease.

Age-associated glial activation and increases in blood-nervous system barrier permeability can increase nervous system iron regionally. Additionally, iron and ferritin levels display age-related increase in cortical, cerebellar, hippocampal, basal ganglia and amygdala astrocytes and microglia (reviewed in Ward et al., 2014). Iron levels displayed linear positive correlation with increasing astrocytic numbers in the murine globus pallidus, striatum and SN, whereas an association between increased iron and microglial numbers was noted in the globus pallidus and striatum (Ashraf et al., 2019). Examining ratios between metal and astrocyte/microglial levels in ageing mice revealed differential alterations between regions. Indeed, whilst iron:astrocytes increased in the SN and globus pallidus of aged (19 and 27 month) mice, striatal iron:astrocytes reduced with ageing. Iron:microglia displayed increases in aged animals across the 3 regions. This might indicate regionally heterogeneous glia-mediated metal dyshomeostasis in ageing. Altogether therefore, specific regions, such as the SN, might be differentially prone to perturbed iron dynamics in both neurons and glia with nervous system ageing, potentially determining regional vulnerability to neurodegenerative disease.

Age-related regional mitochondrial abnormalities might also underly region-specific susceptibility to disease. Analysis of a common mitochondrial DNA (mtDNA) deletion in putamen, cerebellum and cortex human post-mortem tissue (24–94 years) revealed an age-related increase in deletion levels (Corral-Debrinski et al., 1992). Moreover, substantial regional heterogeneity in deletion levels was noted, with the putamen and cortex accumulating more mtDNA deletions than the cerebellum. Another mtDNA deletion revealed similar regional age-related increase. Intriguingly, comparison of post-mortem SN, putamen and frontal cortex mtDNA deletion levels in microdissected neurons indicated that SN neurons were selectively vulnerable to mtDNA deletion accumulation (Bender et al., 2008). Indeed, they possessed higher mtDNA deletion levels than putamen/frontal cortex neurons in AD patients (n = 9; 63–89 years) and age-matched controls (n = 8; 63–83 years), with no differences between disease and control samples. Altogether, it seems that dopaminergic neurons are particularly susceptible to mtDNA deletion accumulation, an age-associated region-selective phenomenon.

Distinct regional alterations in mitochondrial bioenergetics were noted in ageing rats (1–24 months) (Pandya et al., 2016). The brainstem, frontal cortex, cerebellum, striatum and hippocampus were studied, with the hippocampus showing the most diminished mitochondrial bioenergetics at 4, 12 and 24 months, in line with the possibility that this region might be especially prone to brain ageing. Rat cortical, hippocampal and cerebellar mitochondria revealed differential response to elevated calcium during ageing (4, 13 and 25 month age groups) (Brown et al., 2004). More specifically, aged rats displayed increased levels of a fluorescent readout of mitochondrial ROS formation in hippocampal and cortical mitochondria, absent from mitochondria isolated from the cerebellum. Higher rates of ROS formation and larger swelling vs. elevated calcium characterised aged hippocampal and cerebral mitochondria compared to their young counterparts, phenotypes absent from cerebellar mitochondria (Brown et al., 2004). Altogether, distinct regional age-related perturbations in mitochondria might render certain regions prone to toxicity, with the SN and hippocampus highlighted as regions susceptible to such mechanisms. Indeed, mitochondrial dysfunction is an important potential contributory mechanism in AD (reviewed in Cenini and Voos, 2019), PD (reviewed in Bose and Beal, 2016) and ALS (reviewed in Van Damme et al., 2017).

Regional age-associated alterations in calcium buffering might also underlie differential susceptibility to insult. Vulnerable neurons in AD, PD and ALS share the characteristic of possessing low levels of calcium binding proteins (reviewed in Mattson and Magnus, 2006). Mouse knockout of the calcium binding proteins calbindin and parvalbumin, which are downregulated in normal ageing, revealed that calbindin knockout animals had disrupted hippocampus-dependent learning (Moreno et al., 2012). Calbindin knockout animals showed reduced relative cerebral blood volume in the DG and CA1 but not entorhinal cortex, CA3 and subiculum. Decline in the DG but not CA1 was age-related. Parvalbumin knockout animals similarly showed reduced relative cerebral blood volume in the DG and CA1 but this was not age-dependent. Thereby, calbindin loss with ageing might have important implications for age-associated metabolic decline in the hippocampus, with the hippocampal DG/CA1 subregions more sensitive to calbindin decline than the entorhinal cortex. Hence, the hippocampus might be prone to age-related disruption of calcium homeostasis and subsequent metabolic dysfunction. Moreover, whereas ALS-resistant MNs express calcium binding proteins, calbindin and parvalbumin expression is absent in ALS-susceptible MNs, possibly rendering the latter more vulnerable to calcium-mediated toxicity (reviewed in Shaw and Eggett, 2000). Hence the ventral spinal cord, rich in spinal MNs, might be particularly prone to calcium-related aberrance, and calcium dyshomeostasis might have important role in neuronal (reviewed in Mattson and Magnus, 2006) and regional vulnerability to ageing/disease.

Intracellular and/or extracellular aberrant protein aggregation is a hallmark of many neurodegenerative diseases, including AD, PD and ALS. One intrinsic cellular mechanism to remove these aggregates is via proteasome-mediated degradation. In ageing rats (3 weeks-28 months), the multicatalytic proteasome showed differential changes in activity dependent on nervous system region (Keller et al., 2000). Indeed, whilst reduced activity was observed by 3 months in the cortex and hippocampus, by 12 months, multicatalytic proteasome activity was reduced in the hippocampus, cortex and spinal cord. In contrast, the cerebellum and brainstem showed no reduced activity. Age-related region-specific perturbation in proteasome activity might thereby increase the vulnerability of certain regions, such as the hippocampus and spinal cord to damage.

Isolation and sequencing of ribosome-associated mRNA from striatal D1 and D2 spiny projection neurons of young (42 days) and aged (2 years) mice, revealed that aged D1 neurons showed increased sequence reads at/beyond the stop codon, which were not observed in aged D2 neurons or young D1 and D2 samples (Sudmant et al., 2018). Indeed, a set of aged D1 genes showed read coverage limited to the 3’UTR. It was suggested that D1 neurons experienced greater oxidative damage during ageing than D2 neurons. Depletion of ABCE1, a ribosome recycling factor, leads to accumulation of ribosomes near the stop codon and in turn, results in cleavage of the mRNA upstream of the stalled ribosome, yielding 5' and 3' fragments. ABCE1 knockdown in a mouse cell line increased 3’UTR enrichment of certain genes, and exposing ABCE1 to oxidative stress also resulted in enriched 3’UTRs. Together, these data suggest that age-related oxidative stress might impair ABCE1, resulting in ribosome accumulation and subsequent upstream cleavage of the transcript near the stop codon, leading to 3’UTR fragment accumulation in ageing. As evidenced by murine D1 and D2 neurons, this phenomenon seemed cell type-specific; analysis of data from 12 human brain regions revealed 3’UTR accumulation displays region-specific differences. Of the 12 regions, only the cerebellum possessed a negative association between ageing and 3’UTR enrichment, with other regions, including those with higher metabolic activity (and consequently more oxidative stress and a higher vulnerability to neurodegenerative diseases), displaying higher levels of age-associated 3’UTR accumulation. 3’UTRs might also be translated to peptides as a result of oxidative stress (known to increase region-selectively in ageing). Age-related region-specific accumulation of 3’UTRs might lead to structural perturbations, sequestration of key intracellular species and translational aberrancy (Sudmant et al., 2018). Therefore, this important age-associated, region-specific phenomenon might also render certain regions prone to damage, and consequently susceptible to disease.

Regional heterogeneity of synaptic vulnerability in ageing has also been suggested. Similarities in protein expression between young and old occipital cortex synaptic populations in nonhuman primates and humans indicated resistance to age-related damage in this region, whereas the hippocampal synaptic population displayed proteomic heterogeneity on ageing, possibly indicating selective vulnerability of aged hippocampal synapses (Graham et al., 2019). Indeed, resistant (occipital cortex) and vulnerable (hippocampal) brain regions seemed to age differently in nonhuman primates and humans. Potential candidate proteins regulating age-related regional susceptibility were identified, with microglia-associated TGF-β1 revealed as a possible upstream regulator. Notably, TGF-β1 was inhibited in the occipital cortex, again consistent with its activation contributing to regional synaptic susceptibility. The possible microglial-mediated differential vulnerability of synapses to ageing, might render particular regions possessing vulnerable synapses prone to neurodegenerative disease.

Synaptic neurotransmitters might also determine some age and disease-associated susceptibility in certain neuronal populations. Dopamine metabolism, for example, can introduce additional oxidative stress within SN neurons (reviewed in Reeve et al., 2014). More specifically, dopamine oxidative deamination via monoamine oxidases generates H_2_O_2_, which can generate detrimental hydroxyl radicals via iron-associated Fenton chemistry (reviewed in Trist et al., 2019). Other dopamine metabolism pathways can produce superoxide, and dopamine auto-oxidation can also generate ROS. Hence, nervous system dopaminergic regions might be particularly prone to damage. Dopamine is returned to nerve terminals by the dopamine transporter (DAT), after which it is packaged into vesicles (by vesicular monoamine transporter-2; VMAT) as a protective mechanism against dopamine-related metabolic damage (reviewed in Reeve et al., 2014). In young, middle aged and old rhesus monkeys, levels of DAT only showed significant age-associated reduction in the dorsal SN (contrasting the ventral SN and VTA); however, the ventral SN consistently possessed the highest levels of DAT in all age groups (Kanaan et al., 2008). The DAT/VMAT ratio, reflecting the capacity of dopaminergic neurons to accumulate cytoplasmic dopamine, showed no change between young, middle aged and old neurons in the ventral SN; the dorsal SN DAT/VMAT ratio was significantly higher in middle aged than old monkeys. Moreover, in aged animals, the DAT/VMAT was significantly higher in the ventral compared to dorsal SN. Higher DAT levels and higher DAT/VMAT ratios in neurons were associated with increased nitrative damage. It is therefore possible that its dopamine metabolism renders the ventral SN selectively susceptible to age-related damage and disease.

Altogether therefore, differential age-associated alterations to intracellular apparatus, homeostatic mechanisms and important chemical compounds might determine the selective vulnerability of particular regions to damage (Table 3). Thereby, age-related changes at the molecular level might render regions, such as the above-highlighted hippocampus, SNpc and spinal cord, susceptible to neurodegenerative disease.

**Table 3:** Summary: Molecular level determinants of region-specific vulnerability.

Archetypal neurodegenerative disease-associated region	Molecular level mechanisms of regional ageing/susceptibility to neurodegenerative disease	**Key references**
Hippocampus	Iron/ferritin levels increase with age in hippocampal astrocytes/microglia	Ward et al., 2014
	Regionally diminished mitochondrial bioenergetics	Pandya et al., 2016
	Higher mitochondrial reactive oxygen species formation in hippocampal ageing	Brown et al., 2004
	Calbindin knockout mice showed disrupted hippocampal learning and age-related reductions in dentate gyrus relative cerebral blood volume	Moreno et al., 2012
	Regionally reduced activity of the multicatalytic proteasome	Keller et al., 2000
	Hippocampal synapses show proteomic heterogeneity and vulnerability on ageing	Graham et al., 2019

Substantia Nigra	Regional iron accumulation in the ageing substantia nigra	Bilgic et al., 2012
	Regional increase in neuromelanin (which possesses high iron content)	Zecca et al., 2004
	Substantia nigra selectively vulnerable to mitochondrial DNA deletion accumulation	Bender et al., 2008
	Dopamine metabolism associated with oxidative stress/selective vulnerability	Reeve et al., 2014 Kanaan et al., 2008

Spinal Cord	Regionally reduced activity of the multicatalytic proteasome	Keller et al., 2000

## 6 Discussion

Unfortunately, a dearth of disease modifying and life promoting therapies exist for age-related neurodegenerative diseases. Acetylcholine esterase inhibitors and other licenced symptomatic therapies provide some benefit for AD patients, though disease altering treatments do not exist (reviewed in Lane et al., 2018). In PD, there are a number of therapies to aid motor symptoms, including levodopa, however disease progression ultimately impacts long-term treatment efficacy (reviewed in Armstrong and Okun, 2020). Advanced therapy for motor symptoms can involve deep brain stimulation, and approaches attempting to control nonmotor symptoms also exist. Similar to AD, there are no disease modifying drug therapies for PD. The only life promoting drug therapy approved in the UK for ALS treatment is Riluzole, which increases lifespan by approximately 3 months (reviewed in Jaiswal, 2018). A host of supportive and symptomatic therapies exist for ALS, with multidisciplinary team management, optimising nutrition and non-invasive ventilation (in patients requiring respiratory support), providing a survival benefit (reviewed in Hobson and McDermott, 2016), however predicted life expectancy from diagnosis to death remains at 2–5 years. The paucity in disease modifying therapies for neurodegenerative disease patients makes thorough understanding of pathological mechanisms imperative, to ensure optimal bench to bedside translation.

**Fig. 3:**
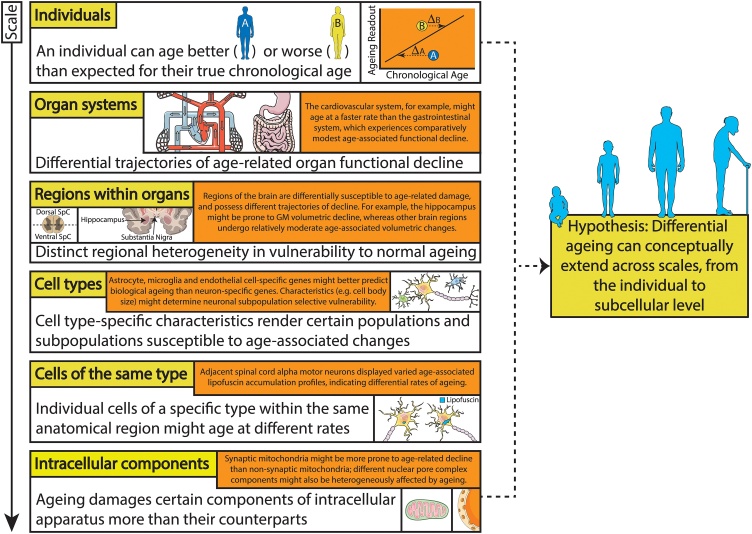
The concept of differential ageing can be applied on an increasingly smaller scale. Differential ageing has been evidenced on an organism level, where an individual can biologically age better or worse than is expected for their chronological age. As well as different individuals ageing differently, ageing affects the function of various organ systems heterogeneously. Moreover, similar principles can be applied to nervous system regions, individual cells and even components within a single cell, which might age disproportionately to their counterparts. Some examples of differential ageing at each scale are provided above, which might have important consequences for age-related disease research. The experimental tractability of analysing nervous system tissue, cellular and molecular differential/delta ageing, as well as its possible future implications therapeutically, are reviewed in the discussion section. GM = grey matter; SpC = spinal cord. Templates used/adapted to create this figure are freely available from Servier Medical Art (https://smart.servier.com/). (References used to create this figure: D’Angelo et al., 2009; Fjell et al., 2013; Jernigan et al., 2001; Khan et al., 2017; Maxwell et al., 2018; Rhinn and Abeliovich, 2017; Soreq et al., 2017; Stauch et al., 2014).

The concept that some individuals age better or worse in biological terms than expected for their chronological age, known as differential/delta ageing, can be applied across scales, with various organ systems, cells and even the intracellular apparatus ageing heterogeneously (Fig. 3). We have reviewed how differential susceptibility of particular regions to age-associated mechanisms at the nervous system tissue, cellular and molecular level might determine archetypal neurodegenerative disease patterns. Indeed, we highlight the hippocampus, SNpc and ventral spinal cord, respectively associated with AD, PD and ALS pathology, as being particularly prone to ageing, and provide an integrated perspective on why age-related neurodegenerative disease pathology is regionally restricted.

Differential/delta ageing quantifies the discrepancy between an individual’s true chronological age and expected biological age derived from an age-associated readout (Rhinn and Abeliovich, 2017). Here, comparison of a phenotypic trait to a cohort-derived regression line based on chronological age ultimately determines the difference between their inferred biological and true chronological age. If one were to scale this concept to a cellular level, one would be comparing an age-associated change in a single cell with a regression line derived from the same cell type from many individuals of varying chronological age. Optimally, one would compare an individual’s ageing readout to an expected value derived from a multi-tissue sample within that individual. For example, rather than using a cohort to determine whether an individual’s hippocampus is ageing better/worse than expected for their chronological age, one would calculate whether an individual’s hippocampus is ageing better/worse than expected from their other brain regions. On a cellular level, one would calculate whether a neuron is ageing better/worse than other types of neuron within one individual, rather than whether a neuron is ageing better/worse than expected using a cohort of individuals. Whether intraindividual multi-tissue/cellular sampling will become experimentally tractable with the development of future technologies remains speculative.

Although we have focussed on three pertinent regions associated with devastating clinical and personal manifestations in AD, PD and ALS, it is possible that the principle of regional disease-susceptibility secondary to their age-vulnerability is a more generalisable principle in disease biology. Indeed, early stage I neurofibrillary alterations in AD arise in the transentorhinal cortex, prior to extending to the entorhinal cortex and hippocampus in stage II (Braak et al., 2006). Similarly, early PD pathology arises in the medulla oblongata and olfactory bulb, with the SNpc involved later in disease progression (reviewed in Armstrong and Okun, 2020). Stage I ALS TDP-43 pathology has been located in the αMNs of the ventral spinal cord, a region discussed in this review, as well as in the agranular motor cortex and the bulbar MNs of cranial nerves V, VII, X, XI, XII (Brettschneider et al., 2013). It would be interesting for future studies to directly compare alterations of these early implicated regions at the nervous system tissue, cellular and molecular level during ageing, with those in the hippocampus, SNpc, ventral spinal cord and other neurodegeneration-resistant nervous system regions, to determine whether they are most prone to ageing, in line with their identification as initial sites of neurodegenerative pathology. Moreover, the principles discussed above might also be applicable to other regionally restricted neurodegenerative diseases associated with ageing, such as Huntington’s disease (reviews: Hou et al., 2019; Machiela and Southwell, 2020). Indeed, some evidence reviewed herein has highlighted the striatum, caudate nucleus, putamen and globus pallidus, as prone to age-related decline, although a comprehensive discussion is beyond the scope of this review. Intriguingly, the caudate nucleus and putamen are selectively susceptible to Huntington’s disease pathology, with the globus pallidus and nucleus accumbens also involved (reviewed in McColgan and Tabrizi, 2018). Future research will determine whether/the extent to which the susceptibility of these regions to ageing determines their vulnerability to Huntington’s disease.

Indeed, the phenomenon of regional susceptibility to disease is not restricted to the nervous system and can be applied to an array of organ systems. The inflammatory bowel diseases, ulcerative colitis and Crohn’s disease, show regional patterns of pathological involvement. More specifically, while ulcerative colitis involvement commences at the rectum with contiguous proximal pathological spread, Crohn’s disease comprises discontinuous pathology throughout the gastrointestinal tract, most frequently involving the terminal ileum or perianal region (reviewed in Khor et al., 2011). It has been hypothesised that the regional vulnerability of the terminal ileum in Crohn’s disease might in part be secondary to genetic factors relating bacterial colonisation and autophagy (reviewed in Caprilli, 2008). Elucidating principles underlying regional selectivity in the nervous system might thereby be transferrable to other organ systems. Regional vulnerability to ageing might also directly apply to other age-related diseases, such as cardiovascular diseases. Intriguingly, the left coronary system is clinically evidenced to be more susceptible to atherosclerosis than the right coronary system, with haemodynamic and geometric mechanisms proposed (reviewed in Chatzizisis et al., 2007). Whether differential susceptibility of left and right cardiac regions to ageing makes some contribution to this regional phenomenon requires further clarification, though is an intriguing hypothesis.

The study of regional ageing in humans is often conducted using post-mortem tissue of deceased subjects at various ages. As well as its inherent limitations (including varied post-mortem interval imposing possible structural tissue changes, often small sample sizes, fixation techniques differentially impacting brain structures (reviewed in Raz and Rodrigue, 2006) and possible subclinical confounding disease), post-mortem ageing studies require cross-sectional design, which poses its own assumptions. While longitudinal studies are possible by ever more sophisticated imaging modalities, such as MRI, neuroimaging techniques also come with limitations. These include possible effects of ageing itself, patient hydration status and operator expertise on image acquisition, as well as both similarities and differences between manual and automated approaches (reviewed in Raz and Rodrigue, 2006). As technology advances and imaging methods evolve, it is possible that neuroimaging might reveal further clarification of the potential intersect between human regional nervous system ageing and disease. With important species differences existing between ageing animal models and humans, such as those evidenced in ageing microglia (Galatro et al., 2017; Pan et al., 2020), integrating orthogonal ageing models is of clear importance for high confidence discovery.

Although not currently viable to robustly address, it is intriguing to postulate whether future methods, including principles described above, might reproduce differential/delta ageing on a molecular level, potentially allowing comparison of the ageing status of different organelles within a single cell. Following similar logic, one could theoretically determine the ageing status of a single organelle. Moreover, similar approaches might allow deeper investigation of the age-susceptibility of cellular subregions, such as comparison of synaptic and dendritic ageing within a single neuron. Although this cannot be comprehensively investigated via current experimental methodology, future studies might unravel differential/delta ageing at the nervous system tissue, cellular and molecular level. In turn, an understanding of differential ageing at each level might reveal mechanisms determining cell type and region-specific vulnerability to ageing.

If indeed regional susceptibility to ageing determines the vulnerability of these same regions to neurodegenerative disease, one can speculate whether targeting a particular aspect of ageing within the human hippocampus, SNpc and ventral spinal cord might have clinical therapeutic viability for AD, PD and ALS respectively. Though principles described here seem possibly less pertinent for oral medication, they might be more relevant for region-specific treatment options, where the most vulnerable nervous system regions must be identified for optimum therapeutic efficacy. Future regional cellular implantation studies in PD for example must optimise where to transplant the cells, along with a number of other considerations (reviewed in Fan and Winanto, 2020). Additionally, a number of antisense oligonucleotide-based therapies are in development or clinical trials for neurodegenerative diseases (reviewed in Bennett et al., 2019). Nervous system penetration by antisense oligonucleotides is an important consideration, with intrathecal administration revealing regional drug penetrance. The highest drug concentrations were noted in the lumbar spinal cord, with lower levels in other spinal cord regions . In the brain, the cortex, hippocampus and cerebellar Purkinje cells possess high concentrations, whilst deep brain structures including the striatum had the lowest concentrations. An understanding of region-specific vulnerability to ageing and neurodegeneration might aid in ensuring the most vulnerable regions are targeted by these treatments, possibly providing the best chance at therapeutic success.

## 7 Conclusions and future perspectives

The effects of normal ageing are intertwined with neurodegenerative disease pathology. Here we review age-related mechanisms at the nervous system tissue, cellular and molecular level which might render particular regions of the brain and spinal cord vulnerable to ageing and neurodegenerative disease. An understanding of regional vulnerability to ageing and disease will guide future development of potential therapeutic strategies targeting nervous system tissue, cellular and/or molecular aspects of differential/delta ageing that are desperately required in devastating neurodegenerative diseases such as AD, PD and ALS (Table 4).

**Table 4:** Summary: Open questions in the field.

Research question		Figure
1	Does regional vulnerability to normal ageing determine region-specific susceptibility in neurodegenerative disease?	1
2	Do mechanisms of ageing at the nervous system tissue, cellular and molecular level determine regional vulnerability to both normal ageing and neurodegenerative disease?	2
3	Can differential ageing conceptually extend across scales, from the individual to subcellular level?	3
4	Are the initial sites affected by neurodegenerative pathology also those that are most prone to ageing?	–
5	Can the concept of regional susceptibility to ageing be extended across other age-related diseases, such as cardiovascular disease?	–
6	Is it experimentally tractable to directly compare the ageing status of single cells, cellular subregions, or individual organelles within cells?	–
7	How can an understanding of region-specific vulnerability to ageing and disease be used to guide therapeutic advance e.g. ASO delivery, cellular implantation?	–

## Funding

V.A.P. is funded by the 10.13039/501100000833Rosetrees Trust [548644] and the University College London MBPhD Programme. R.P. holds an MRC Senior Clinical Fellowship [MR/S006591/1].

## CRediT authorship contribution statement

**Virenkumar A. Pandya:** Conceptualization, Funding acquisition, Methodology, Visualization, Writing - original draft, Writing - review & editing. **Rickie Patani:** Conceptualization, Funding acquisition, Methodology, Supervision, Writing - review & editing.

## Declaration of Competing Interest

The authors declare no conflict of interest.
